# An Under-Recognized Sequela of Myocardial Infarction: Lipomatous Metaplasia

**DOI:** 10.7759/cureus.10560

**Published:** 2020-09-20

**Authors:** Matthew S Campbell, Alper Duran, Cihan Duran

**Affiliations:** 1 Radiology, University of Texas Medical Branch, Galveston, USA; 2 Radiology, Mount Sinai West, New York, USA; 3 Radiology, McGovern Medical School at The University of Texas Health Science Center, Houston, USA

**Keywords:** t1 with and without fat suppression, lipomatous metaplasia, diagnostic techniques for lm, clinical significance of lm

## Abstract

Lipomatous metaplasia is an infrequently discussed condition characterized by adipose tissue replacing scar tissue from ischemic events. Lipomatous metaplasia specifically of the myocardium is an adverse physiological result of myocardial infarction. In the past, several different imaging and diagnostic techniques were utilized to recognize lipomatous metaplasia of the myocardium. The aim of this study is to discuss an individual case with this condition to highlight several key aspects that are under-discussed in current literature, such as determining the most suitable modality for the recognition of lipomatous metaplasia.

## Introduction

The clinical significance of lipomatous metaplasia remained unclear for a long time owing to the relatively little amount of literature about the topic in the past. More recently, however, there have been a few studies in the literature highlighting the association between lipomatous metaplasia and significant adverse cardiac conditions such as ventricular arrhythmia, infarct size, and heart failure [1]. However, even with this important association, there is still a scarcity of literature on lipomatous metaplasia. Furthermore, there is also still a lack of consensus on the most efficient imaging and diagnostic techniques for recognizing lipomatous metaplasia. Hence, this case is being reported in order to provide more insight into the clinical significance of this condition and to enhance the understanding about the most effective radiological diagnostic techniques used for recognizing lipomatous metaplasia.

## Case presentation

We present a case of left ventricular (LV) lipomatous metaplasia after myocardial infarction evaluated with cardiac magnetic resonance imaging. A 56-year-old male with anterior myocardial infarction eight years ago presented with a transient ischemic attack. The patient underwent a transthoracic echocardiogram as part of the diagnostic evaluation, which suggested possible LV thrombus. He was referred to cardiac MRI to assess for possible LV thrombus and to quantify the LV function.

**Figure 1:**
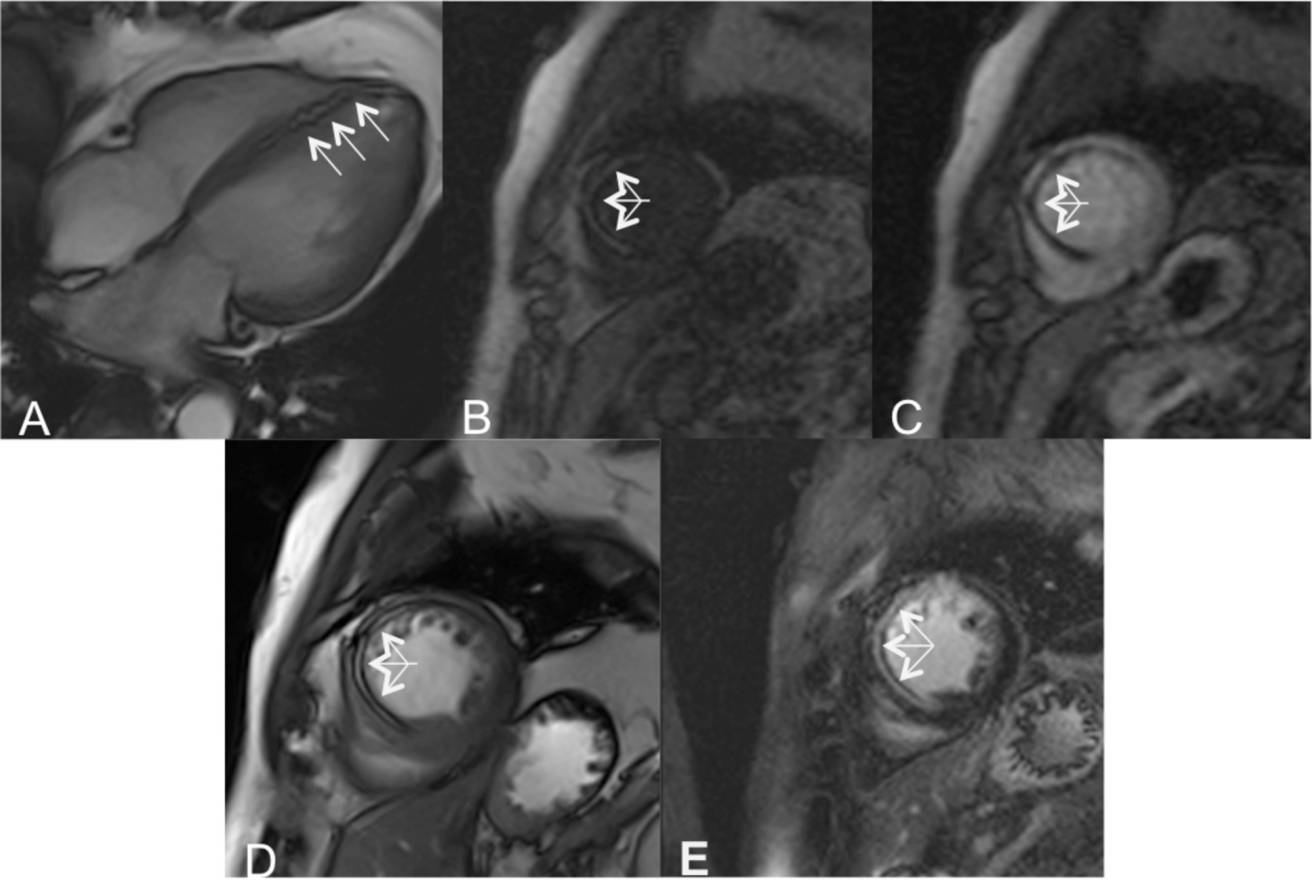
Imaging of lipomatous metaplasia A linear high signal lesion with peripheral low signal ‘etched’ appearance due to chemical shift artifact (arrows) in four-chamber and short-axis balanced SSFP in the mid anteroseptum consistent with lipomatous metaplasia (A, D). T1-weighted short-axis gradient-echo image demonstrates high signal intensity (arrows) in the mid-anterior-septal segment (B). A severe first-pass perfusion defect is seen (arrows) in the corresponding myocardial segments following the administration of gadolinium (C). The late gadolinium enhancement (LGE)-balanced SSFP image shows a high signal, which is due to fatty metaplasia rather than fibrosis (E) SSFP: steady-state free precession

Original cardiac MRI showed moderately impaired LV systolic function with hypokinesis of the mid to apical anteroseptal wall. There was a linear high signal lesion with surrounding low signal chemical shift artifact in balanced steady-state free precession (SSFP) cine sequences in the mid anteroseptum, mid anterior, and apical septal segments (Figures 1A, 1D). T1-weighted gradient-echo images demonstrated corresponding high signal intensity consistent with lipomatous metaplasia (Figure 1B). There were severe first-pass myocardial perfusion defects in the corresponding myocardial segments (Figure 1C). The patient declined to proceed with suggested cardiac angiography or any further cardiac intervention. The patient was continued on beta-blockers and statins and was discharged.

## Discussion

Following a myocardial infarction, myofibroblasts lay connective tissue that serves as a protective agent against future myocardial rupture and prevents dilation of the infarcted area [2]. However, in the case of lipomatous metaplasia, the myofibroblasts instead differentiate into adipose tissue through mechanisms that are still poorly understood [2]. Given the findings in various studies and this case report, the most efficient modality for diagnosis of lipomatous metaplasia is T1-weighted cardiac MRI with and without fat suppression [3,4]. Recent studies have elucidated the clinical significance of lipomatous metaplasia as a significant predictor of hospitalization due to heart failure, all-cause mortality, and sustained ventricular arrhythmia in patients with chronic ischemic heart disease [1]. Additionally, the presence of lipomatous metaplasia was shown to have a greater adverse effect on cardiac remodeling and larger infarctions overall [1]. Because lipomatous metaplasia has been shown to be a strong predictor of these issues, the clinical significance of this phenomenon is clear. The lipomatous metaplasia can appear hyperechoic on the echocardiogram and can be mistaken for thrombus as in our patient, which is why determining the best imaging techniques for this condition is crucial. Despite the high prevalence of ischemic heart disease, lipomatous metaplasia is still rarely reported. This may be due to a lack of consensus among studies on the clinical significance of lipomatous metaplasia in the past [5]. Alternatively, it could be due to a lack of capability to properly characterize myocardial adipose tissue by the more common imaging modalities such as echocardiography, and the relatively limited usage of technologies like MRI and cardiac CT. Although multi-slice CT has been used to detect lipomatous metaplasia of the infarcted myocardium, cardiac MRI is the preferred imaging modality. This is because of its high tissue contrast, and due to its advantage in the evaluation of myocardial viability in the context of ischemic heart disease [1].

## Conclusions

The bright signal in T1-weighted images without fat suppression and high signal lesion with chemical shift artifact in balanced SSFP sequences show lipomatous metaplasia without intravenous contrast media. Therefore, cardiac MRI, with its high contrast resolution capabilities and lack of ionizing radiation, is the most suitable modality to identify lipomatous metaplasia of the myocardium.
